# Elucidation of the 3D Structures of two Human RNA Triple Helices using Cryo-EM

**DOI:** 10.1063/4.0001102

**Published:** 2025-10-27

**Authors:** Madeline M. Mousseau, Conner J. Langeberg, Jeffrey S. Kieft, Jessica A. Brown

**Affiliations:** 1 Department of Chemistry and Biochemistry, University of Notre Dame, Notre Dame, IN, USA; 2 Innovative Genomics Institute, University of California, Berkeley, CA, USA; 3 New York Structural Biology Center, New York, NY, USA

## Abstract

Metastasis-associated lung adenocarcinoma transcript 1 (MALAT1) is an abundant nuclear long non-coding RNA (lncRNA) with a 3′-end triple helix, protecting the RNA from degradation. Multiple endocrine neoplasia β (MENβ) is another eukaryotic lncRNA that has a predicted triple helix structure, but this triple helix has not been structurally validated. Using cryogenic-sample electron microscopy (cryo-EM), we determined the structures of the two similar RNA triple helices fused to the Tetrahymena (TetP6b) scaffold. The MALAT1 triple helix-dTetP6b was globally resolved at 3.1 Å and the MENβ triple helix-dTetP6b at 3.7 Å, confirming the formation of the MENβ triple helix. The RNA triple helices both exhibited local resolutions of 4.2-8.7 Å, likely caused by increased flexibility as observed in 3D variability analysis. These lower local resolutions prevent de novo modeling of the MENβ triple helix. AlphaFold 3 was employed to predict the 3D structure of the MENβ triple helix. The AlphaFold 3 structure results in alternative binding interactions that differ from the MALAT1 triple helix at the lower triplex-duplex junction. UV thermal denaturation assays of MALAT1 and MENβ triple helix mutants will provide insights into key structural features for the respective triple helices. In addition, structural elements flanking the triple helices aid in the stable formation of the respective triple helix. This study elucidated the structure of two eukaryotic lncRNA triple helices, providing further knowledge and validation of naturally occurring RNA triple helices and targeted therapies for these two disease-associated lncRNAs.

